# Gait Asymmetry Post-Stroke: Determining Valid and Reliable Methods Using a Single Accelerometer Located on the Trunk

**DOI:** 10.3390/s20010037

**Published:** 2019-12-19

**Authors:** Christopher Buckley, M. Encarna Micó-Amigo, Michael Dunne-Willows, Alan Godfrey, Aodhán Hickey, Sue Lord, Lynn Rochester, Silvia Del Din, Sarah A. Moore

**Affiliations:** 1 Institute of Neuroscience/Institute for Ageing, Newcastle University, Newcastle Upon Tyne NE4 5PL, UK; christopher.buckley2@newcastle.ac.uk (C.B.); maria.mico-amigo@newcastle.ac.uk (M.E.M.-A.); sue.lord@aut.ac.nz (S.L.); lynn.rochester@ncl.ac.uk (L.R.); silvia.del-din@newcastle.ac.uk (S.D.D.); 2 EPSRC Centre for Doctoral Training in Cloud Computing for Big Data, Newcastle University, Newcastle Upon Tyne NE4 5PL, UK; m.dunne-willows@newcastle.ac.uk; 3 Department of Computer and Information Science, Northumbria University, Newcastle upon Tyne NE1 8ST, UK; alan.godfrey@northumbria.ac.uk; 4 Department of Health Intelligence, HSC Public Health Agency, Belfast BT2 7ES, Northern Ireland; Aodhan.Hickey@hscni.net; 5 Auckland University of Technology, 55 Wellesley St E, Auckland 1010, New Zealand; 6 The Newcastle upon Tyne Hospitals NHS Foundation Trust, Newcastle Upon Tyne NE7 7DN, UK; 7 Institute of Neuroscience (Stroke Research Group), Newcastle University, 3-4 Claremont Terrace, Newcastle upon Tyne NE2 4AE, UK; 8 Stroke Northumbria, Northumbria Healthcare NHS Foundation Trust, Rake Lane, North Shields, Tyne and Wear NE29 8NH, UK

**Keywords:** stroke, asymmetry, accelerometer, gait, trunk, reliability, validity

## Abstract

Asymmetry is a cardinal symptom of gait post-stroke that is targeted during rehabilitation. Technological developments have allowed accelerometers to be a feasible tool to provide digital gait variables. Many acceleration-derived variables are proposed to measure gait asymmetry. Despite a need for accurate calculation, no consensus exists for what is the most valid and reliable variable. Using an instrumented walkway (GaitRite) as the reference standard, this study compared the validity and reliability of multiple acceleration-derived asymmetry variables. Twenty-five post-stroke participants performed repeated walks over GaitRite whilst wearing a tri-axial accelerometer (Axivity AX3) on their lower back, on two occasions, one week apart. Harmonic ratio, autocorrelation, gait symmetry index, phase plots, acceleration, and jerk root mean square were calculated from the acceleration signals. Test–retest reliability was calculated, and concurrent validity was estimated by comparison with GaitRite. The strongest concurrent validity was obtained from step regularity from the vertical signal, which also recorded excellent test–retest reliability (Spearman’s rank correlation coefficients (rho) = 0.87 and Intraclass correlation coefficient (ICC_21_) = 0.98, respectively). Future research should test the responsiveness of this and other step asymmetry variables to quantify change during recovery and the effect of rehabilitative interventions for consideration as digital biomarkers to quantify gait asymmetry.

## 1. Introduction

Hemiparesis after stroke typically results in reduced walking speed, an asymmetrical gait pattern, and a reduced ability to make gait adjustments that consequentially limit community ambulation and physical activity [1,2,3,4]. Reduction in both predisposes an already at risk population to further cardiometabolic disease [5,6]. Therefore, the improvement of gait is a worthwhile and common target for interventions after stroke. Gait asymmetry, if not addressed early in the recovery process, can prolong and increase gait impairment due to compensatory mechanisms, leading to an increasingly asymmetric gait pattern [7]. The latter is inefficient and requires increased energy expenditure. Consequently, falls risk increases, further reducing levels of physical activity [8]. In order to quantify asymmetry and its improvement from targeted rehabilitative interventions, it is essential to have both valid and reliable tools that are able to quantify movement quality/compensatory strategies of the whole body during gait.

Tests such as the 10 m walk [9] and scales such as the Dynamic Gait Index [10] are used to measure gait after stroke. Although useful and practical for application to clinical settings, these tests are susceptible to subjectivity and not specifically designed to capture the cardinal symptoms of gait after stroke, such as asymmetry. Instrumented walkways can objectively measure asymmetry and have shown excellent intra and inter-rater reliability in subacute stroke [11]. Practically, they are costly and need a controlled dedicated environment with a trained specialist to operate; therefore, they are mainly limited to research settings [12]. From a biomechanical perspective, they limit the number of steps collected per trial and solely obtain information of the participant’s footfall. They are not designed to measure the movement of the whole body, where synergistic compensatory movement strategy information may be quantified such as compensatory movements of the pelvis [8,13]. Traditionally, gaining this information would rely on three-dimensional motion analysis systems. However, due to the even higher cost, required experience, and time to use relative to instrumented mats, their application is also limited to research settings [12]. Therefore, a need exists for a valid tool that is capable of quantifying whole body asymmetry, while also being feasible for routine clinical adoption.

Wearable accelerometers are a relatively low-cost alternative that are capable of measuring human movement from a variety of contexts while capturing parameters that are difficult to quantify from clinical inspection by the human eye [1,14]. Previous attempts to quantify measures of asymmetry indicative of spatiotemporal information of the feet with accelerometers have shown their feasibility, but also poor concurrent validity with reference standards of Gaitrite [1]. Therefore, the development of algorithms to capture the complex nature of asymmetry post-stroke has been encouraged [1]. Numerous asymmetry variables exist that have been obtained from cyclical acceleration signals during gait such as variables derived from the frequency domain [15,16]. These variables vary according to the complexity of the sensor, the number of sensors used, their location, and the population on which they were tested [17,18,19]. Relative to the discreet spatiotemporal movement of the feet equivalents, variables quantifying asymmetry from the cyclical signals of the lower back better classified post-stroke gait from controls [16,18,20]. Their advantage stems from considering the acceleration as a complete waveform, not neglecting temporal information outside of the time domain, which may enable a more complete description of the signal and a better characterisation of gait post-stroke [17].

Previously, studies quantifying asymmetry from acceleration signals of the trunk during post-stroke gait typically focus on differences from a control group, adopt a minimal data set of variables, and to our knowledge do not report the concurrent validity or reliability to reference standards. Knowledge of the most robust asymmetry variables that are capable of quantifying similar information to reference standards using clinically feasible tools is important to further the field. This study compares the validity and test–retest reliability of a wide range of novel acceleration-derived variables to quantify asymmetry post-stroke from a single sensor located on the trunk.

## 2. Materials and Methods

### 2.1. Study Design and Setting

This cross-sectional study was undertaken in the gait laboratory at the Clinical Ageing Research Unit, Campus for Ageing and Vitality, Newcastle upon Tyne, UK.

### 2.2. Participants

The study was approved by the Greater Manchester West Research and Ethics Committee (NRES Committee Northwest-Greater Manchester West 15/NW/0731). All subjects gave informed written consent for the study according to the Declaration of Helsinki.

Inclusion criteria: Community-dwelling stroke survivor; at least one month post-stroke onset; mild to moderate gait deficit defined by clinical observation of gait asymmetry including reduced stance time, increased swing time in the affected limb and/or reduced gait speed/balance problems; no changes in gait-related ability over the past month based on self-report and able to walk 10 m with/without a stick.

Exclusion criteria: Medical problems other than stroke impacting on gait e.g., osteoarthritis. Participants were recruited via advertisement or therapist referral. All eligible participants were consecutively invited to participate in the study.

### 2.3. Demographic and Clinical Measures

The following data were collected at baseline: age, gender, height and weight, date of stroke, stroke type (Oxford Community Stroke Project Classification [21]), stroke impairment (National Institute of Health Stroke Scale [22]), presence of hemiplegia (clinical observation by two independent experienced clinicians), walking stick use, ankle foot orthosis (AFO) use.

### 2.4. Test Protocol

**Figure 1:**
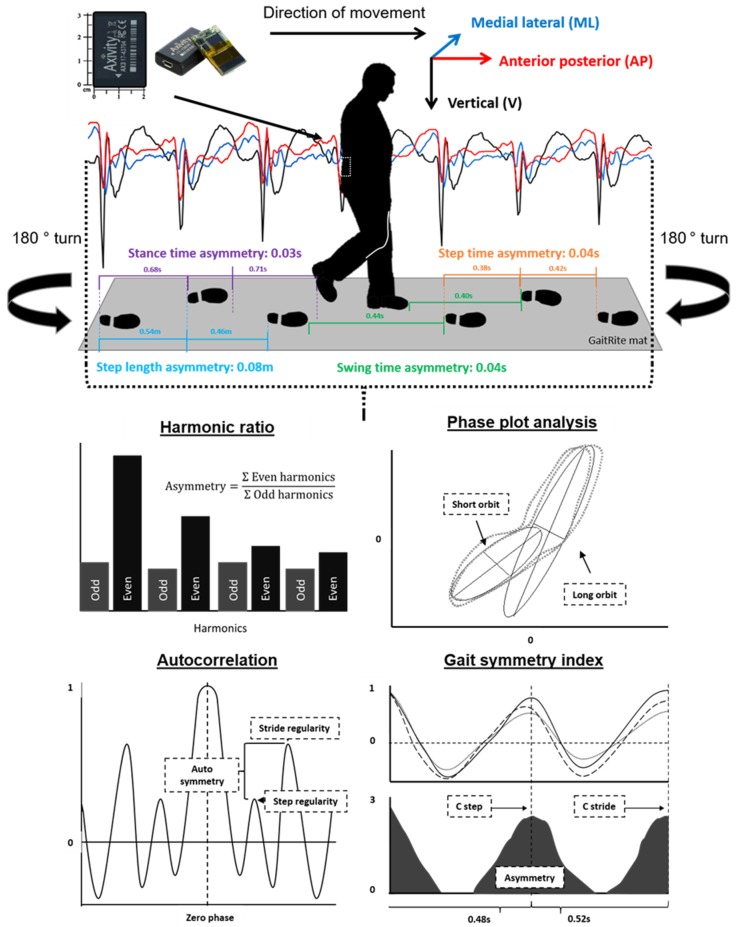
Indication of the instrumentation and the protocol used to collect the acceleration signal and the asymmetry parameters from the GaitRite mat. Also pictured is the acceleration-derived asymmetry variables and the means for the calculation of asymmetry following the processing of the raw acceleration signal.

Participants were asked to walk at their preferred pace in a straight line for 4 × 10 m intermittent trials (see Figure 1). The trials were repeated on two occasions (Time 1 and Time 2) one week apart (±2 days). A GaitRite instrumented walkway was positioned in the walk path (dimensions were 7.0 m × 0.6 m, spatial accuracy of 1.27 cm and temporal accuracy of one sample (240 Hz, ~4.17 ms) (GaitRite: Platinum model GaitRite, software version 4.5, CIR systems, NJ, USA)). The participants wore an AX3 wearable sensor located at their fifth lumbar vertebrae (L5). The AX3 is a single tri-axial accelerometer-based wearable (AX3, Axivity, York, UK https://axivity.com/, cost ≈ £100, dimensions 23.0 mm × 32.5 mm × 7.6 mm). The AX3 weighs 11 g and has a memory of 512 Mb. AX3 data capture occurs with a sampling frequency of 100 Hz (16-bit resolution) at a range of ±8 g. Recorded AX3 accelerations were stored locally on the device’s internal memory and downloaded upon the completion of each session.

### 2.5. Asymmetry Variables

Acceleration-derived asymmetry variables were selected based upon their ability to represent levels of asymmetry from signals measured from a single accelerometer located at the trunk. The variables that were selected as representative of asymmetry were the harmonic ratio [16], autocorrelation [20], gait symmetry index [18], and phase plot analysis [23,24,25] (described in more detail below). Four spatiotemporal variables extracted from GaitRite were selected as measures of asymmetry as defined by Lord et al. [26]. The spatiotemporal asymmetry variables included step time asymmetry, stance time asymmetry, swing time asymmetry, and step length asymmetry, and these were calculated as the absolute difference between consecutive left and right steps.

### 2.6. Description of Acceleration-Derived Variables

All data analysis relating to the raw acceleration signals was performed using MATLAB (version 9.4.0, R2018a). For a full description for the algorithm and data segmentation techniques applied to the accelerometer data, please see references [27,28]. In brief, the vertical acceleration underwent continuous wavelet transformation to estimate the initial contact and final contact in the gait cycle [28]. To ensure that the steady-state gait was analyzed, the initial and final three steps were removed from the signal. Prior to the calculation of additional variables, the acceleration signals were realigned to the earth’s gravitational constant [29,30] and a low-pass Butterworth filter with a cut-off frequency of 20 Hz. A full description of the following variables and the required algorithms is the supplied by the provided references. Additionally, they have been summarised in Appendix A.

#### 2.6.1. Harmonic Ratio

The harmonic ratio (HR) describes the step-to-step symmetry within a stride from calculating a ratio of the odd and even harmonics of a signal following fast Fourier transformation [16,31]. This method has been shown previously to reflect increased asymmetry for those post-stroke relative to age and speed-matched controls [16].

#### 2.6.2. Autocorrelation

The unbiased autocorrelation was also calculated due to its ability to reflect the step and stride regularity and the symmetry between the two (autocorrelation symmetry) [20,32,33]. Previously, it has been shown as better capable to characterise hemiplegic gait relative to footfall variables [20,32].

#### 2.6.3. Gait Symmetry Index

The gait symmetry index (GSI) is a more recently proposed variable, which was calculated based upon the concept of the summation of the biased autocorrelation from all three components of movement and a subsequent calculation of step and stride timing asymmetry [18]. It has been shown to be more sensitive than and highly correlated with levels of asymmetry measured with two sensors located at the feet of participants post-stroke [18].

#### 2.6.4. Phase Plot Analysis

Phase plot analysis (aka Poincaré analysis) was performed on vertical components of the acceleration signal [23,24,25]. This method has had previous applications within electrocardiogram studies. It works by plotting periodic signals as a function of their past values. The resulting ellipses or orbits and the properties thereof can then assess asymmetries in the associated gait. Phase plot analysis also offers the ability to assess intra step correlation i.e., the correlation of signals from immediately successive step cycles, which necessarily corresponds to left-versus-right asymmetry.

#### 2.6.5. Measures Indicative of Stability

Although not indicative of asymmetry, the root mean square of the acceleration signal (Acc RMS) and also its first time derivative (Jerk RMS) were calculated for their potential to highlight synergistic compensatory strategies during gait post-stroke [13,16]. Their test–retest reliability needs to be established in the literature.

### 2.7. Statistical Analysis

Analysis was completed using SPSS v25 (IBM). The normality of data was tested with a Shapiro–Wilk test. Descriptive statistics (median and interquartile range) were calculated for gait characteristics measured by AX3 and GaitRite. Concurrent validity between the AX3 acceleration-derived variables and those of the GaitRite at Time 1 were tested using Spearman’s rank correlation coefficients (RHO). For the AX3 acceleration-derived variables, the test–retest reliability between Time 1 and 2 was established using Spearman’s rank correlation coefficients (RHO), intraclass correlation coefficient (ICC_21_), and limits of agreement (LoA) expressed as a percentage of the mean of the two variables and the 95% LoA. For all analyses, statistical significance was set at *p* < 0.05. Predefined acceptance ratings for ICC_21_ were set at excellent (≥900, 0.0%–4.9%), good (0.750–0.899, 5.0%–9.9%), moderate (0.500–0.749, 10.0%–49.9%), and poor (50.0%) [1,34]. The selection for the most robust variable was based upon the variable with the highest Spearman rank correlation coefficient with the asymmetry variable obtained from the GaitRite while also recording an ICC_21_ greater than 0.8 for test–retest reliability.

## 3. Results

Twenty-five participants were recruited to the study. Data for two participants who wore a fixed plastic AFO were removed from the analysis, because individual data analysis (including video observations) revealed that the step detection applied were not appropriate for these two participants due to a lack of possible plantar flexion. This was not the case for the remaining participants, as the video analysis confirmed the step detection algorithm was effective to detect both heel strike and toe off [1]. Demographic information for the remaining 23 participants is displayed in Table 1.

### 3.1. Concurrent Validity of the Asymmetry Variables

**Figure 2:**
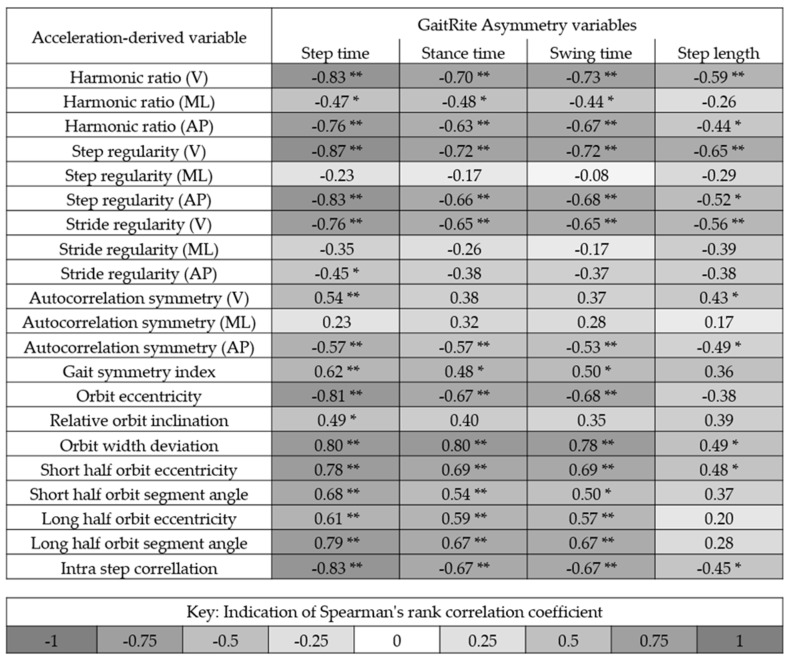
Indication of the correlation between the asymmetry variables quantified using a GaitRite mat and the variables proposed to measure asymmetry from the acceleration signals from the trunk. Black indicates a strong positive or negative correlation. * and ** denotes significance at the 0.05 and 0.01 level, respectively. V = Vertical acceleration, ML = Medial lateral acceleration, and AP = Anterior posterior acceleration.

Figure 2 shows the correlation between the asymmetry variables quantified using a GaitRite mat (step time asymmetry, stance time asymmetry, swing time asymmetry, and step length asymmetry) and the acceleration-derived variables proposed to measure asymmetry. Overall, step time asymmetry correlated most with the acceleration-derived variables. Step regularity (vertical acceleration) had the highest concurrent validity with step time asymmetry (−0.87). Six other variables had high levels of agreement (+0.80) (HR V, step regularity (V), step regularity (AP), orbit eccentricity, orbit width deviation, and intra step correlation). Five correlated with step time asymmetry and orbit width deviation correlated with stance time asymmetry. The smallest correlations were achieved by the outputs of the autocorrelation from the medial lateralcomponent of the signal and also a variety of the outputs from the phase plot analysis.

### 3.2. Test–Retest Reliability of the Variables

Table 2 demonstrates the test–retest reliability between the wearable variables measured one week apart (Time 1 versus Time 2). The most reliable variables were step regularity (V) and HR (V), both recording an ICC_21_ of 0.98. Taken from the ICC_21_ values, excellent reliability was achieved for 12 out of the 27 variables tested. These came from the majority of autocorrelation outputs except for step regularity (ML), stride regularity (AP), and autocorrelation symmetry (vertical acceleration (V) and medial lateral acceleration (ML)) direction, the GSI, the HR in the V and AP direction, Jerk RMS, and the short half-orbit segment angle form the phase plot analysis. Good reliability was achieved for a further five variables (stride regularity (AP), autocorrelation symmetry (V), relative orbit inclination, short half orbit eccentricity, and long half orbit eccentricity).

### 3.3. Selection of the Most Robust Variable

Table 3 highlights the variables that best correlated with spatiotemporal gait variables calculated from GaitRite while also achieving an ICC_21_ greater than 0.8 for test–retest reliability. For the GaitRite variables of asymmetry, step regularity (V) achieved the highest concurrent validity due to its correlation with step time asymmetry (RHO = 0.87 and ICC_21_ = 0.98 **). The second highest concurrent validity was the HR in the vertical direction, which correlated with swing time asymmetry (RHO = 0.73 and ICC_21_ = 0.98 **).

## 4. Discussion

This study examined the concurrent validity and reliability of a comprehensive range of asymmetry variables derived from a single accelerometer located on the trunk and identified step regularity as the most robust outcome. Step regularity showed strong concurrent validity and excellent test–retest reliability when compared with GaitRite outcomes reflecting asymmetry. This contrasts with previous work based on the AX3 sensor, which achieved poor to moderate criterion validity (Spearman’s rank correlation coefficient of RHO = 0.01 to 0.601) for variables engineered to replicate spatiotemporal asymmetry variables calculated from GaitRite [1]. Although clinically more challenging to interpret than traditional spatiotemporal variables, our results support the adoption of novel variables to quantify asymmetry as robust digital variables for measuring asymmetrical gait post stroke.

With one exception (HR correlation with swing time asymmetry), variables calculated from performing an autocorrelation procedure on the original acceleration signal were more strongly correlated with GaitRite asymmetry. Hodt–Billington and colleagues [20] found that autocorrelation variables taken from the trunk were better at discriminating gait post-stroke from controls relative to GaitRite variables of asymmetry. The strength of the autocorrelation procedure may stem from analysing continuous successive steps. Complex measures such as gait asymmetry are not simply portrayed within a single discreet gait cycle; this concept has been highlighted before, whereby continuous measures have been described to highlight different asymmetry causes, symptoms, and gait strategies such as particular compensatory techniques [17]. Data from our study indicate that participants with high asymmetry produced poor forward propulsion from the affected limb, instead of relying on the more dominant limb to achieve progression at the end of each stride. This can be observed by the lack of step regularity and its diminution relative to stride regularity in the AP, ML, and V directions, replicating the gait strategy described by Balasubramanian et al. [35]. The autocorrelation method is well designed to reflect this synergistic gait strategy, which might explain the high correlation found from this sample of participants. However, this strategy will likely vary among a broader range of participants and throughout recovery. Other methods may better reflect true levels of asymmetry at different stages of recovery from acute, early subacute, late subacute, and chronic stroke, meaning that they should still be considered as potential variables [17,20].

Previously, Iosa et al. [16] assessed symmetry together with upright gait stability post-stroke and showed that relative to speed-matched controls, higher instabilities (Acceleration RMS) and reduced symmetry of trunk movements (as measured using the HR) were recorded. In this study, HR in the vertical direction was the only HR variable that performed favourably to autocorrelation variables due to its correlation with swing time asymmetry (RHO = −0.73) while also recording excellent reliability (ICC_21_ = 0.98). Since we did not assess control subjects, we could not determine the best measure to characterise gait post-stroke and highlight the compensatory mechanisms adopted relative to healthy controls. This is a broader aim for ongoing work. However, it has been previously highlighted that compensation strategies may be beneficial to increase gait ability, but this occurs at the compromise of stability. Thus, variables such as Acceleration and Jerk RMS should always be considered in addition to variables directly linked to asymmetry, aiming to provide a more holistic description of gait patterns [13,16]. Future research should explore this relationship so that a holistic, multivariate wearable approach can better assess gait strategies during recovery post-stroke. This potentially would quantify what movements are beneficial to gait, while also highlighting the impact of compensation strategies, consequently quantifying separate movements that can be targeted for rehabilitation.

Although previously suggested as a variable representative of asymmetry in stroke [18], the GSI performed relatively poorer to the previously discussed variables, despite also being based on the autocorrelation (biased) of accelerometry. This was unexpected, as GSI theoretically is designed to detect the asymmetry within temporal footfall parameters. Equally, the autocorrelation symmetry variables did not perform better than step regularity alone, despite being designed to the capture the difference between step and stride regularity and therefore the symmetry between them. Potentially, the GSI and the autocorrelation symmetry did not quantify the synergistic movement strategy that the step regularity variable was suited to highlight and the reason for its favourable concurrent validity. The GSI and the autocorrelation symmetry variables may be better suited to highlight different compensatory synergies at different stages of recovery such as during acute, early subacute, late subacute, and chronic stages, and therefore should not be neglected in future research.

Select phase plot variables achieved RHO values greater than 0.8 when compared to GaitRite asymmetry values and also demonstrated good to excellent reliability, therefore highlighting their ability to quantify symmetry post-stroke. Adaption to the algorithms to the other directional components other than vertical and comparison with controls would better test their application as a biomarker. Similar to the other variables capable of quantifying movements in the AP and ML direction, there is the possibility that they can highlight a new domain of asymmetry separate from the asymmetry footfall asymmetry variables captured by GaitRite. Future research should explore this upper and lower body relationship post-stroke to examine the similarities and differences during gait and determine if added value is obtained [36,37].

All data were collected in a controlled environment; however, wearable technology is not limited by the testing environment and for improved ecological validity; obtaining data from the participant’s community is desired [38]. To this goal, future research should utilise the variables tested in the laboratory in the participant’s free-living environment. For free-living gait, the majority of walking bouts for people with Parkinson’s disease and older adults have been found to be below 10 s, and it has been inferred that these bouts are when the participants are indoors [39]. One limitation with autocorrelation is that it relies on successive steps in a straight line. For free-living data, variables such as the HR may be more useful during these short walking bouts due to their ability to be calculated from a single stride in addition to successive steps [31,40]. Future research should assess the ability of these variables to accurately and reliably quantify asymmetry during short walking bouts or if tested refined spaces, as for this population, the median (and interquartile range) bout length was 16.3 (6.2) seconds for data collected over seven days [1].

### 4.1. Limitations

The relatively small sample size and limited heterogeneity with respect to time post-stroke did not allow us to determine what variables are the best at quantifying asymmetry for a more general sample or recovery stage-specific populations [41]. Future work is required on a larger sample size that ranges in time since stroke to discover what variables are the most capable to perform as objective biomarkers over all stages of recovery as one variable may not be appropriate for all, and compensatory strategies may change between the different stages of stroke recovery. Equally, future research should confirm that these results are replicable with different accelerometers with differing sampling frequencies, ranges, and resolutions. Further limitations stem from the reliance of the step detection algorithm. Data from two participants was not analysed due to their use of a fixed AFO that impacted on heel strike and the performance of the algorithm, which was based on the detection of initial and final contact within the gait cycle. Future research should integrate/develop step detection algorithms for participants requiring fixed AFOs to broaden application. Alternatively, the variables should be developed so that the cyclical nature of a signal may divide gait cycles (similar to the method used for phase plots) as opposed to methods that rely on detecting the initial and final contact of the foot.

### 4.2. Applications

These results provide evidence that asymmetry can accurately and reliably be calculated using a single accelerometer. Although much work is needed for accelerometers to be routinely adopted [42,43], these results give evidence that asymmetry can be objectively quantified using a tool applicable for many purposes. Consequently, the variables tested here may then act as a digital biomarker to quantify the impact of targeted interventions proposed to improve gait timing mechanisms and gait asymmetry (e.g., auditory rhythmical cueing) [44]. Accelerometers provide a potentially low burden method for clinicians to collect data from a variety of environments, increasing the ability to objectively quantify asymmetry during stroke rehabilitation. Alongside application within the clinic, accelerometer data can be collected on gait asymmetry in naturalistic environments, thus removing the Hawthorn effect/observer bias associated with clinical testing. With increased development, these variables may provide continuous asymmetry focussed feedback for self-progress specific to each participant during rehabilitation.

## 5. Conclusions

Gait asymmetry after stroke can be measured robustly using a single wearable sensor on the trunk. Step regularity is the most valid and reliable asymmetry outcome, which is quantified by performing autocorrelation on the vertical component of the signal. The variables tested performed favourably to previous studies that also used GaitRite as the reference. Consequently, their adoption, in addition to other wearable-derived spatiotemporal variables of gait, are encouraged as they provide a more holistic description of gait that appears to indicate compensatory movement post-stroke. Future research is encouraged on larger populations where asymmetry is expected, during recovery/interventions to identify which wearable variables are biomarkers for gait asymmetry and compensatory mechanisms during gait. This will allow for increased accuracy in determining effective interventions.

## Figures and Tables

**Table 1:** Participant characteristics.

**Demographics (n = 23)**	
Gender (male/female)	19/4
Age (years)	63 ± 11
Body mass index	26 ± 4
**Stroke characteristics**	
Time since stroke (months)	66 ± 48 (range 5–201)
**Stroke subtype (OCSP)**	
Total anterior circulation	11
Partial anterior circulation	6
Lacunar	3
Posterior circulation	3
**Stroke impairment**	
NIHSS score (0–40)	4 ± 3 (range 0–11)
NIHSS lower limb score (0–4)	1 ± 0.7 (range 0–3)
Walking speed (m/s)	0. 9 ± 0.4
Marked hemiplegia (Yes/No)	15/8
Walking aid (number (%))	3 (13%)
Push Aequi ankle foot orthosis (number (%))	4 (17%)

Where appropriate mean and standard deviation are displayed, OCSP (Oxford community Stroke Project), NIHSS (National Institute for Health Stroke Scale).

**Table 2:** Test–retest reliability (one week apart) for acceleration-derived variables.

Variables	Median (IQR)		Agreement			
	T1	T2	Median Difference (%)	ICC_21_	LOA % (95% LoA)	Rho
Harmonic ratio (V)	1.71 (1.37)	1.70 (1.23)	−0.01	0.98 **	1.94 (2.52, 1.36)	0.92 **
Harmonic ratio (ML)	1.38 (0.60)	1.57 (0.72)	0.14	0.71 **	1.56 (2.80, 0.31)	0.71 **
Harmonic ratio (AP)	1.26 (0.97)	1.39 (0.92)	0.10	0.92 **	1.54 (2.34, 0.73)	0.91 **
Step regularity (V)	0.53 (0.47)	0.52 (0.54)	−0.02	0.98 **	0.51 (0.67, 0.34)	0.96 **
Step regularity (ML)	0.42 (0.20)	0.44 (0.18)	0.04	0.73 **	0.44 (0.69, 0.19)	0.61 **
Step regularity (AP)	0.51 (0.43)	0.40 (0.49)	−0.20	0.92 **	0.37 (0.68, 0.07)	0.87 **
Stride regularity (V)	0.70 (0.25)	0.68 (0.27)	−0.03	0.94 **	0.66 (0.85, 0.46)	0.88 **
Stride regularity (ML)	0.59 (0.14)	0.66 (0.20)	0.12	0.93 **	0.57 (0.78, 0.37)	0.73 **
Stride regularity (AP)	0.74 (0.18)	0.75 (0.13)	0.01	0.87 **	0.70 (0.92, 0.48)	0.74 **
Autocorrelation symmetry (V)	0.53 (0.26)	0.52 (0.29)	0.56	0.80 **	0.18 (0.40, −0.03)	0.76 **
Autocorrelation symmetry (ML)	0.10 (0.19)	0.16 (0.25)	0.09	0.59 *	0.19 (0.44, −0.05)	0.49 *
Autocorrelation symmetry (AP)	0.18 (0.15)	0.19 (0.14)	0.61	0.93 **	0.36 (0.62, 0.10)	0.79 **
Gait symmetry index	0.21 (0.37)	0.35 (0.43)	−0.02	0.92 **	0.47 (0.70, 0.23)	0.82 **
Orbit eccentricity	7.79 (6.27)	8.32 (15.13)	0.00	0.72 **	0.97 (1.04, 0.91)	0.70 **
Relative orbit inclination	0.01 (0.01)	0.01 (0.01)	0.07	0.76 **	11.02 (28.02, −5.99)	0.60 **
Orbit width deviation	0.01 (0.02)	0.00 (0.02)	−0.07	0.66 **	0.01 (0.05, −0.02)	0.65 **
Short half orbit eccentricity	5.32 (6.35)	4.12 (5.31)	−0.38	0.73 **	0.02 (0.07, −0.03)	0.87 **
Short half orbit segment angle	0.02 (0.05)	0.01 (0.04)	−0.23	0.95 **	7.74 (15.28, 0.20)	0.57 **
Long half orbit eccentricity	5.20 (10.73)	5.61 (6.55)	−0.16	0.79 **	0.04 (0.13, −0.05)	0.59 **
Long half orbit segment angle	0.89 (0.41)	0.88 (0.20)	0.08	0.45	7.77 (26.32, −10.78)	0.57 **
Intra step correlation	1.05 (0.04)	1.05 (0.04)	−0.01	0.58 *	0.78 (1.29, 0.28)	0.68 **
Acceleration RMS (V)	0.18 (0.09)	0.17 (0.06)	0.00	0.03	1.03 (1.24, 0.83)	0.41
Acceleration RMS (ML)	0.25 (0.15)	0.24 (0.15)	−0.06	0.90 **	0.17 (0.24, 0.10)	0.68 **
Acceleration RMS (AP)	8.53 (8.00)	8.57 (7.47)	−0.04	0.20	0.26 (0.62, −0.10)	0.21
Jerk RMS (V)	6.29 (4.18)	6.36 (4.15)	0.01	0.96 **	9.32 (13.49, 5.14)	0.93 **
Jerk RMS (ML)	6.22 (4.89)	6.42 (6.88)	0.01	0.97 **	7.39 (10.67, 4.11)	0.90 **
Jerk RMS (AP)	1.71 (1.37)	1.70 (1.23)	0.03	0.96 **	7.26 (11.23, 3.28)	0.92 **

* and ** denotes significance at the 0.05 and 0.01 level, respectively. V = Vertical acceleration, ML = Medial lateral acceleration, and AP = Anterior posterior acceleration, RMS = root mean square.

**Table 3:** Indication of what wearable sensor variable recorded the highest Spearman’s rank correlation coefficient with each variable obtained by the GaitRite mat. The Spearman’s rank correlation coefficient between the two devices and the intraclass correlation coefficient is displayed for each variable.

	GaitRite Variable	Acceleration Derived Variable	Spearman’s Rank Correlation Coefficient (RHO)	ICC_21_ (Test–Retest)
Asymmetry	Step time (s)	Step regularity (V)	0.87	0.98 **
	Swing time (s)	Harmonic ratio (V)	0.73	0.98 **
	Stance time (s)	Step regularity (V)	0.72	0.98 **
	Step length (m)	Step regularity (V)	0.65	0.98 **

** denotes significance at the 0.01 level. V = Vertical acceleration.

**Figure A1:**
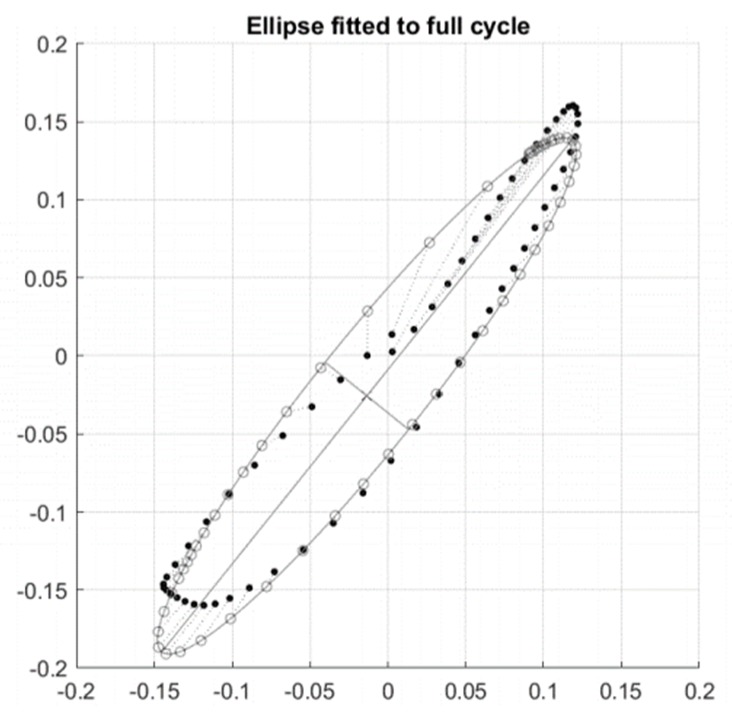
A single orbit with a fitted conic (ellipse).

**Figure A2:**
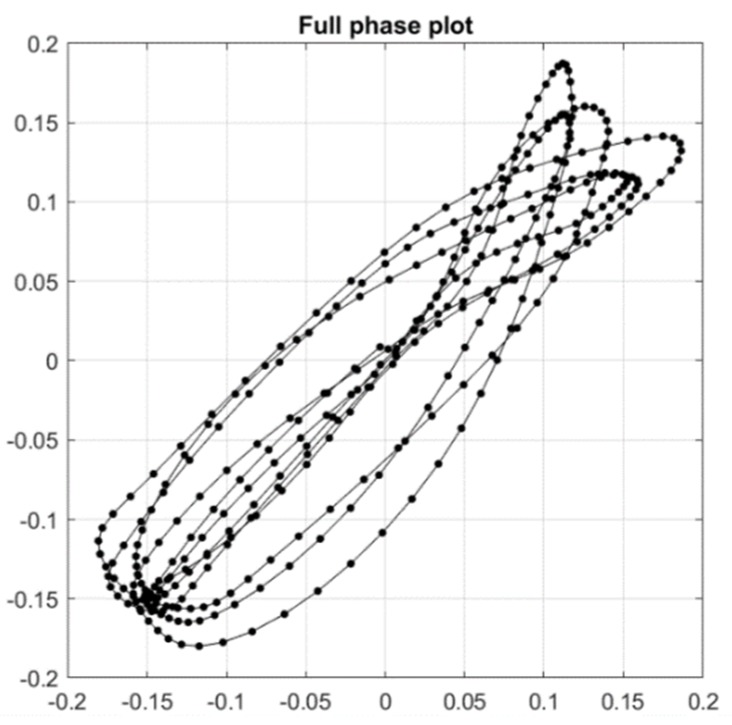
Complete phase plot comprising 7 continuous gait cycles.

**Figure A3:**
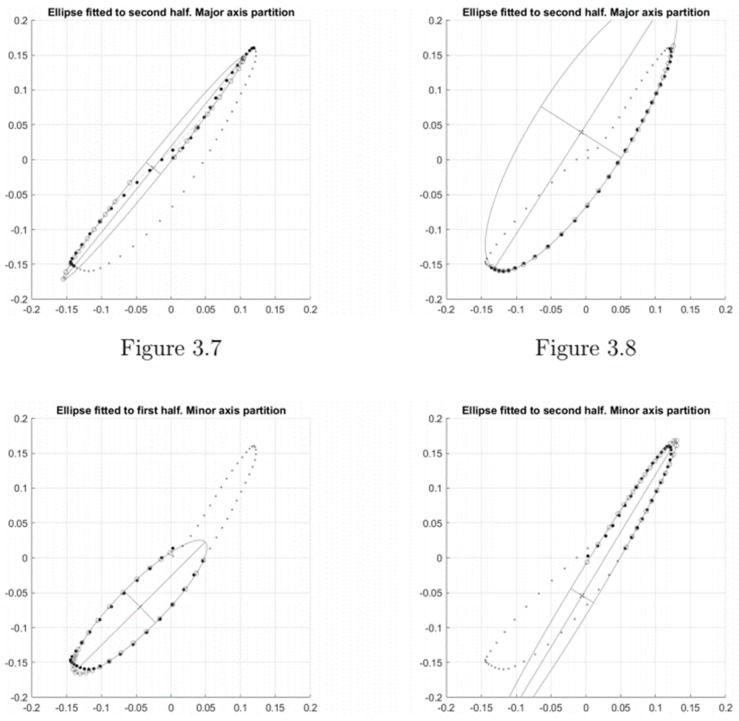
Indication of the different conic (ellipses) fitted to the major/minor axis and the first/second halves.

## Data Sharing

Data cannot be shared publically but can be available upon reasonable request to the corresponding author, as per local data sharing policies.

## Appendix A

### Appendix A.1. Acceleration-Derived Variable Definitions

**Table A1:** Indication for the variables used from the signal-derived variables and their respective definitions.

Variable	Definition
Harmonic ratio (V, ML, AP)	The step-to-step symmetry within a stride from calculating a ratio of the odd and even harmonics of a signal following fast Fourier transformation.
Step regularity (V, ML, AP)	Estimated as the normalized unbiased autocovariance for a lag of one step time. Thus, this feature reflects the similarity between subsequent steps of the acceleration pattern over a step. Values of this feature close to 1.0 (maximum possible value) reflect repeatable patterns between subsequent steps.
Stride regularity (V, ML, AP)	Estimated as the normalized unbiased autocovariance for a lag of one stride time. Thus, this feature reflects the similarity between subsequent strides of the acceleration pattern over a stride cycle.
Autocorrelation symmetry (V, ML, AP)	Difference between step and stride regularity designed to quantify the level of symmetry between them and indicative of symmetry during a straight walk.
Gait symmetry index	Calculated based upon the concept of the summation of the biased autocorrelation from all three components of movement and a subsequent calculation of step and stride timing asymmetry.
Orbit eccentricity (V)	Average eccentricity of all fully fitted ellipses.
Relative orbit inclination (V)	Average angle subtended by alternating fitted ellipses within a bout of gait.
Orbit width deviation (V)	Standard deviation of minor axes lengths of all fully fitted ellipses. Analogous to Principle Component Analysis (second component).
Short half orbit eccentricity (V)	Difference in eccentricity of two ellipses fitted to each half-cycle of a full orbit in the phase plot. Averaged over all orbits in a bout’s phase plot.
Short half orbit segment angle (V)	Difference in inclination of two ellipses fitted to each half-cycle of a full orbit in the phase plot. Averaged over all orbits in a bout’s phase plot.
Long half orbit eccentricity (V)	Difference in eccentricity of two ellipses fitted to each half-cycle of a full orbit in the phase plot. Averaged over all orbits in a bout’s phase plot.
Long half orbit segment angle (V)	Difference in inclination of two ellipses fitted to each half-cycle of a full orbit in the phase plot. Averaged over all orbits in a bout’s phase plot.
Intra step correlation (V)	Average correlation of acceleration signal corresponding to step i with that of step i-1. I.e., a lag-1 autocorrelation where a single lag is one step cycle’s duration.
Acceleration RMS (V, ML, AP)	The calculation of the root mean square of the acceleration signal.
Jerk RMS (V, ML, AP)	The calculation of the root mean square of the first time derivative of the acceleration signal (jerk).

### Appendix A.2. Explanation and Equation for Each Acceleration Derived Variable for Asymmetry

#### Appendix A.2.1. Harmonic Ratio

The harmonic ratio is a measure based upon the premise that a stride contains two steps and therefore, during continuous walking, accelerations should repeat in multiples of two. The variable quantifies how well these accelerations are repeated in each stride compared to when accelerations do not repeat and are therefore out of phase. Therefore, the ratio of in and out-of-phase accelerations is a measure of how symmetric the participant is walking. To calculate the harmonic ratio, it is required to evaluate the harmonic content of the acceleration signal using the stride frequency from the analysis of frequency components. Following a fast Fourier transform (using the FFT function in MATLAB), a ratio be can created from the first 20 harmonics extracted from the Fourier series. Due to the AP and V components of the signals being biphasic, the ratio for these components is determined by the sum of the even harmonics (in phase movement) divided by the sum of the odd harmonics (out-of-phase movement).

$${\mathrm{HR}}_{\mathrm{AP},\;V}\;=\;\frac{\Sigma \;\mathrm{Amplitudes}\;\mathrm{of}\;\mathrm{even}\;\mathrm{harmonics}}{\Sigma \;\mathrm{Amplitudes}\;\mathrm{of}\;\mathrm{odd}\;\mathrm{harmonics}}$$

For the ML component of the signal due to only showing only one dominant acceleration peak within a stride cycle (whereby the odd harmonics are in-phase and even harmonic out-of-phase), the opposite is performed.

$${\mathrm{HR}}_{\mathrm{ML}}\;=\;\frac{\Sigma \;\mathrm{Amplitudes}\;\mathrm{of}\;\mathrm{odd}\;\mathrm{harmonics}}{\Sigma \;\mathrm{Amplitudes}\;\mathrm{of}\;\mathrm{even}\;\mathrm{harmonics}}$$

As a gait measure, a higher harmonic ratio indicates a better symmetry between steps within a single stride For the AP and V components.

#### Appendix A.2.2. Autocorrelation

Autocorrelation is calculated taking the complete signal of the time when the participant was in contact with the GaitRite mat. Plots of an autocorrelation estimate are used to inspect the structure of a cyclic component within a time series. To do this, the generic unbiased autocorrelation function of the sample sequence x(i) was computed using the below equation:

$$\mathrm{Ad}\left(m\right)\;=\frac{1}{N-\left|m\right|}\overset{N-\left|m\right|}{\underset{i=1}{\sum }}x\left(i\right)\cdot x\left(i+m\right)$$

where N is the number of samples and m is the time lag expressed as number of samples.

Since phase shifts can be performed with identical results in both positive and negative directions relative to the original time series, an autocorrelation plot is conventionally organized symmetrically with the zeroth shift located centrally. This central value was used to normalize the signal so that its maxima was one. For a time series of trunk accelerations during walking, autocorrelation coefficients can be produced to quantify the peak values at the first and second dominant period, representing phase shifts equal to one step and one stride, respectively (see Figure 1 as an example). A tailored MATLAB code was used to detect these peaks, particularly using the signals power density to determine the windows in which the peaks would occur. For the symmetry between the step and stride regularity, the absolute difference was calculated as a measure of asymmetry instead of the ratio, which is more conventionally used. This was because the between-step and between-stride autocorrelations may approach zero if the regularity between neighboring steps or neighboring strides is low.

#### Appendix A.2.3. Gait Symmetry Index (GSI)

Differently from the aforementioned autocorrelation measures, the gait symmetry index (GSI) uses a second-order Butterworth low-pass filter with the cut-off frequency of 10 Hz to filter the complete time series and then uses the biased version of the autocorrelation function as displayed below:

$$\mathrm{Ad}\left(m\right)\;=\frac{1}{N}\sum_{i=1}^{N-\left|m\right|}x\left(i\right)\cdot x\left(i+m\right).$$

The maximum time lag was 4 s (400 samples), which approximates 2.5 times a single stride duration in post hemiplegic stroke patients. This window length was chosen to capture the repetition of stride cycles in very slow walking. A coefficient of stride cycle repetition (Cstride) was the sum of the positive autocorrelation coefficients of the three axes as a function of the equation displayed below:

Cstride(t) = ADv(t) + ADml(t) + ADap(t); if AD(t) <0, AD(t) = 0.

The coefficient of step repetition (Cstep) was the norm of autocorrelation coefficients as a function of the equation displayed below:

$$\mathrm{Cstep}\left(t\right)\;=\;\sqrt{\mathrm{ADv}\left(t\right)+\mathrm{ADml}\left(t\right)+\mathrm{ADap}\left(t\right)};\;\mathrm{if}\;\mathrm{AD}\left(t\right)<0,\;\mathrm{AD}\left(t\right)=0.$$

One stride time (Tstride) equals t when the Cstride had the maximum value. The hypothesis was that in a perfect symmetric gait pattern, two consecutive steps have the same step duration of 0.5 × Tstride. Thus, the maximum value of Cstep was set at √3 when the autocorrelation coefficient of each acceleration axis was 1 at zero-lag (t = 0). The gait symmetry index (GSI) was Cstep (0.5 × Tstride) normalized to its value at zero-lag, as indicated in the below equation:

$$\mathrm{Cstep}\left(t\right)=\mathrm{Cstep}\left(0.5*\mathrm{Tstride}\right)/\sqrt{3}.$$

#### Appendix A.2.4. Phase Plot Analysis

To create an ellipse to apply the following models, the vertical acceleration signal was first transformed to a horizontal–vertical coordinate system and filtered with a low-pass fourth order Butterworth filter at 20 Hz. Following piecewise integration, the full vertical excursion signal must be restored via concatenation of the resultant integrals. Here, the phase shift is introduced. We restore two such vertical excursion signals, one of which is exactly one step cycle lagged behind the other i.e.,:

PP1(tt)=PP0(tt−nn)

where *n* is the number of data points comprising a step interval in the vertical excursion signal and *PP*1 and *PP*0 are the lagged and original vertical excursion signal, respectively.

The following conic model is fitted to the two-dimensional phase plot data. This fitting is performed on each orbit in turn.

$$ax^{2}+by^{2}+cxy+dx+ey+f=0$$

In the case of ellipse fitting to phase plot data, x and y are taken to be PP1 and PP0.

The above model defines an ellipse subject to the following constraint.

$$c^{2}-4ab<0$$

This constraint is used to ensure that an elliptical conic is fitted to the data as opposed to a hyperbola or parabola. The model defined by the conic equation can be fitted using ordinary least squares to find an estimate of $\hat{A}=\;\left(\hat{a},\;\hat{b},\;\hat{c},\;\hat{d},\;\hat{e}\right).\;f$ is set equal to 1 to avoid a trivial solution.

The above form of an ellipse does not lend itself well to geometric interpretation, so the following parameterisation is implemented:

$$\frac{{\left(x-g\right)}^{2}}{r{\;}_{1}^{2}}+\;\frac{{\left(y-k\right)}^{2}}{r{\;}_{2}^{2}}=1.$$

However, this form does not account for inclined ellipses. To account for the significant inclination of ellipses, the following rotated coordinate system is introduced:

$$x^{\prime }=\left(x-g\right)\cos\left(\theta \right)+\left(y-k\right)\sin\left(\theta \right)$$

$$y^{\prime }=\left(y-k\right)\mathrm{ycos}\left(\theta \right)+\left(x-g\right)\sin\left(\theta \right).$$

This form of ellipse and rotated coordinate system ensure more straightforward interpretation of the ellipses and more intuitive feature extraction.

This Figure A1 shows one such ellipse fitted to a single orbit of a phase plot. From this ellipse, we can extract features relating to the eccentricity and inclination. In general, phase plots consist of many orbits and their respective fitted ellipses (Figure A2). Further features can be extracted by assessing the relative inclination of ellipses from alternating orbits. In general, these inclinations oscillate about the value $\theta =\frac{\pi }{4}\;$.

Features extracted from ellipses fitted to entire orbits are considered primary features. Ellipses can be fitted to partial orbits; for example, two separate ellipses can be fitted to both halves of an orbit where the orbit in question is halved according to its major/minor axes. This leads to four additional ellipses fitted to each orbit of a phase plot (Figure A3). As an example, take the two ellipses fitted to either half of the shown orbit following halving via the minor axis (Figure A3, lower two figures). Features are extracted from these ellipses by extracting their relative characteristics e.g., their inclination relative to the other, the ratio of their areas, etc. Features extracted from ellipses fitted to partial orbits in this way are considered secondary phase plot features.
